# Characterization and Optimization of Fermentation Conditions of *Roseateles* sp. L2-2, a Novel Chitin-Degrading Bacterium from the Intestine of *Odorrana margaretae*

**DOI:** 10.3390/microorganisms13092033

**Published:** 2025-08-30

**Authors:** Yanmei Cai, Xinyu Li, Shuang Chen, Qichao Liu, Hongxiang Lu, Jiahui Xie, Wei Li, Guiying Chen

**Affiliations:** 1 College of Life Sciences, Sichuan Normal University, Chengdu 610101, China; caiyanmei@stu.sicnu.edu.cn (Y.C.); zsbcst@outlook.com (X.L.); 2022090305@stu.sicnu.edu.cn (S.C.); 2022090636@stu.sicnu.edu.cn (Q.L.); 2022090325@stu.sicnu.edu.cn (H.L.); 2 The Faculty Geography Resource Sciences, Sichuan Normal University, Chengdu 610101, China; xiejh@stu.sicnu.edu.cn

**Keywords:** *Odorrana margaretae*, gut microbiota, polyphasic taxonomy, chitin degradation, functional genomics, fermentation optimization

## Abstract

Microorganisms with chitin-degrading capabilities play a crucial role in the biological control of crop pests and diseases as well as in the treatment of organic waste. In this study, a chitin-degrading bacterium, designated L2-2, was isolated from the intestine of *Odorrana margaretae* collected in Mount Emei, Sichuan, China. Based on physiological and biochemical characteristics, 16S rRNA gene sequencing, and phylogenetic analysis of 31 conserved housekeeping genes in the whole genome, strain L2-2 was identified as a member of the genus *Roseateles*, named *Roseateles* sp. L2-2. This strain is able to grow on agar medium with colloidal chitin as the sole carbon source and form clear hydrolysis zones. After optimizing fermentation conditions (including concentrations of nitrogen and carbon sources, culture time, and pH), the enzyme activity was increased to 3.46 U/mL, which was 24 times higher than the initial enzyme activity. Functional genome annotation showed that the strain contains genes encoding endochitinases of the GH18, GH23, and GH46 families, as well as genes encoding β-glucosidases of the GH1, GH2, GH3, and GH109 families, indicating its genetic basis for chitin-degrading potential. This study expands the diversity of known chitin-degrading bacteria and provides a promising microbial resource for the bioremediation of chitinous waste and sustainable pest control in agriculture.

## 1. Introduction

Chitin is a linear polysaccharide composed of N-acetyl-D-glucosamine (GlcNAc) units linked by β-1,4-glycosidic bonds. It is the second most abundant natural biopolymer on Earth, surpassed only by cellulose [1]. Chitin is widely found in the structural components of various organisms, including the cell walls of fungi and insects, the endoskeletons of mollusks, and the exoskeletons of crustaceans [2]. At least millions of tons of chitin waste are generated globally each year. As a major producer of aquatic resources, China also generates large quantities of chitin-containing waste [3]. Chitinases, classified under glycoside hydrolases (GHs), are capable of hydrolyzing the β-1,4-glycosidic bonds between GlcNAc units in chitin [4]. Based on their catalytic mechanisms, chitinases are generally categorized into endochitinases and exochitinases, with the latter further subdivided into chitobiosidases and β-1,4-glucosidases. Endochitinases cleave chitin randomly at internal sites to produce soluble chitin oligosaccharides, whereas exochitinases release GlcNAc monomers from the non-reducing ends of the polymer chains [5].

Based on amino acid sequences and structural characteristics, chitinases are categorized into several glycoside hydrolase (GH) families, with the most prominent being GH18, GH19, and GH20, and additional members classified into GH23 and GH46 [6]. This taxonomic diversity highlights the broad spectrum of biological functions these enzymes perform across various taxa. Within these families, GH18 chitinases have the widest distribution, found in microorganisms, plants, and animals, making them a central focus in chitinase research. Bacteria are key natural decomposers of chitin and play crucial roles in chitin turnover and nutrient cycling in both terrestrial and marine ecosystems. Current research into chitin-degrading bacteria primarily focuses on their isolation and identification, optimization of fermentation parameters, and applications in managing agricultural pests and diseases. As a green alternative to chemical pesticides, chitinases offer significant potential for the development of environmentally friendly biocontrol strategies [7].

Several chitin-degrading bacteria have been isolated from diverse ecological niches, including tidal flats [8], freshwater lakes [9], the feces of Goeldi’s monkey (*Callimico goeldii*) [10], the gut of the Asian common toad (*Duttaphrynus melanostictus*), and the European Sea Bass (*Dicentrarchus labrax*) [11,12]. *Bacillus paralicheniformis* [13], *Bacillus thuringiensis* [14], and *Serratia marcescens* [15] are among the common bacteria with high chitinase activity. Atheena et al. increased chitinase yield to 13.46 U/mL—2.62 times higher than the baseline—by optimizing fermentation parameters for their isolated strain [16]. Sonia Sharma and colleagues improved chitinase production from strain S167, isolated from soil samples, by 48-fold through culture optimization [17]. Similarly, Luo et al. achieved a 26-fold increase in enzyme activity (up to 91.80 U/mL) after optimizing fermentation conditions for strain C9, a chitinase-producing *Streptomyces* isolated from semi-arid Algerian soil [18]. In terms of agricultural applications, the chitinase produced by *Serratia marcescens* strain SEN retains high activity under alkaline conditions and significantly increased the mortality of *Spodoptera litura* larvae when added to their diet [19]. Furthermore, Li et al. demonstrated that strain LY03 can attach to algal surfaces via flagella, secrete chitinase to disrupt algal cell walls, and exhibit broad-spectrum algicidal activity [20]. Mount Emei, located in southwestern China, is home to a rich diversity of amphibians and serves as an important biological resource bank in China. Frogs are highly dependent on chitin-containing prey in their diet, such as flies and locusts; chitin is one of the components of the exoskeletons of these insects. The intestinal tract, as the main digestive site, harbors a large number of microbial communities. Based on the strong interaction between the microbiota and the environment, frogs become a reasonable and efficient host choice for isolating chitin-degrading bacteria. This study aimed to isolate chitin-degrading bacteria from the intestine of *Odorrana margaretae* inhabiting Emei, Sichuan. It was identified as a member of the genus *Roseateles* through polyphasic taxonomy, named *Roseateles* sp. L2-2, which provides a new perspective for the ecological distribution and functional diversity of the genus.

*Mitsuaria* (*Mit.su.a’ri.a*. N.L. fem. n. *Mitsuaria*, referring to Matsue City, the origin of the soil samples from which the type strain was isolated) is a genus of Gram-negative, strictly aerobic, non-spore-forming, rod-shaped bacteria. The cells measure approximately 0.7–1.0 μm in width and 2–4 μm in length, and exhibit motility via a single polar flagellum. They test positive for oxidase and catalase activities. The type species of the genus is *Mitsuaria chitosanitabida* [21]. In 2022, Liu et al. conducted a comprehensive taxonomic revision based on genomic, phylogenetic, and phenotypic evidence, resulting in the reclassification of five genera, including *Mitsuaria* and *Roseateles*, into a unified genus—*Roseateles* [22]. As of April 2025, the genus *Roseateles* comprises 14 validly published species listed in the LPSN database of the International Committee on Systematics of Prokaryotes (https://lpsn.dsmz.de/genus/Roseateles, accessed on 20 April 2025). Members of this genus are all Gram-negative, motile, rod-shaped bacteria that do not form endospores. Intracellular granules of poly-β-hydroxybutyrate (PHB) are commonly observed as storage materials, and reproduction occurs via binary fission. The predominant respiratory quinone is ubiquinone-8 (UQ-8), and the type species is *Roseateles depolymerans* [23].

To date, strains of the *Roseateles* genus have been isolated from a wide range of ecological environments, including freshwater [24,25], legume root nodules [26], well water samples [27], soil [28], industrial wastewater, and water for hemodialysis [29]. However, there have been no reports of *Roseateles* strains isolated from animal gastrointestinal tracts.In this study, a strain, L2-2, which is an inducible chitinase-producing bacterium belonging to the genus *Roseateles*, was first isolated from the intestinal tract of amphibians. The strain was identified using polyphasic taxonomic methods, and its general phenotypic, physiological, biochemical characteristics as well as genomic properties were described. Meanwhile, the genetic basis of its chitin-degrading ability was revealed through whole-genome analysis. Furthermore, the fermentation conditions for its enzyme production were optimized. According to the optimal fermentation conditions (ammonium sulfate addition of 3.092 g/L, culture time of 4.4 days, and pH of 7.1), the enzyme activity after optimization was increased to 3.46 U/mL (The chitinase activity before optimization was 0.1394 U/mL), which was 24 times higher than the initial enzyme activity. This study provides a theoretical basis and technical support for enriching the diversity of the genus *Roseateles*, expanding the resources of chitin-degrading bacteria, and their applications in agriculture and environmental governance.

## 2. Materials and Methods

### 2.1. Isolation of Chitin-Degrading Bacterium

Samples were collected from an green odorous frog (*Odorrana margaretae*) individual near Qingyin Pavilion, Emei Mountain, Sichuan Province, China. The collection time was 17 October 2022, at the geographical coordinates 103°23′40.2″ E, 29°34′30″ N. After the samples were brought back to the laboratory, dissection was performed under sterile conditions. The intestinal contents were collected and inoculated onto chitin screening medium, then placed on a shaker for enrichment culture with shaking at 30 °C and 180 rpm for 3–4 days. (Animal ethics approval documents from relevant institutions have been obtained). All the reagents used were purchased from Aladdin (Shanghai, China), the screening medium (1 L) consists of: neutral colloidal chitin (5%) 10 g, FeSO_4_∙7H_2_O 0.01 g, MnCl_2_ 0.01 g, ZnSO_4_ 0.01 g, MgSO_4_∙7H_2_O 0.5 g, tryptone 1 g, KH_2_PO_4_ 0.3 g, K_2_HPO_4_ 0.7 g, NH_4_Cl 1 g, and agar powder 18 g (for solid medium). The pH of the medium is adjusted to 7.2, and it is sterilized at 121 °C for 20 min. Preparation of 5% colloidal chitin (40 mL): Take 2 g of chitin powder into a 500 mL Erlenmeyer flask, add 96 mL of concentrated hydrochloric acid, and seal the flask. Shake at 37 °C and 150 rpm for 1 h until the chitin is fully dissolved. Then, place it in a 4 °C refrigerator for 24 h. Add 280 mL of absolute ethanol to precipitate for 5 h, and colloidal chitin will precipitate out. Collect the precipitate by centrifugation, wash it repeatedly with distilled water until neutral, adjust the volume to 40 mL, and store it at 4 °C for later use.

A small piece of thigh muscle tissue, roughly the size of a pea, was preserved in anhydrous ethanol for morphological identification. Additionally, a portion of the tissue was fixed in 10% formalin and labeled appropriately for archiving as a reference specimen. Following enrichment, the bacterial suspension underwent serial dilutions and was plated onto solid chitin agar medium. The plates were incubated at 30 °C. Initially, six chitin-degrading bacterial strains were screened based on their ability to form transparent zones. After isolation and purification, colonies that produced distinct transparent zones with a diameter greater than 10 mm on the chitin medium were chosen for further purification. This process was carried out for seven generations in all cases, ensuring the isolation of single colonies. Ultimately, a strain with robust chitin-degrading capabilities was obtained, designated as L2-2 (with a transparent zone diameter exceeding 13 mm), and preserved in 20% glycerol at −80 °C for long-term storage.

### 2.2. Phylogenetic Analysis Based on 16S rRNA Gene

The 16S rRNA gene of strain L2-2 was amplified using universal primers 27F (5′-AGAGTTTGATCCTGGCTCA-3′) and 1492R (5′-GGTTACCTTGTTACGACTT-3′) [30]. The PCR cycling conditions were as follows: pre-denaturation at 94 °C for 5 min; denaturation at 94 °C for 30 s; annealing at 55 °C for 30 s; extension at 72 °C for 1.5 min; and a final extension at 72 °C for 10 min. The middle three steps (denaturation, annealing, and extension) were repeated for 30 cycles. The PCR product was sequenced and submitted to the NCBI GenBank database. Homology analysis was conducted using BLAST (version 2.14.0, Basic Local Alignment Search Tool) on 20 March 2024, and closely related reference strains were selected. The 16S rRNA gene sequences of related strains were downloaded from the NCBI database and aligned using MEGA (version 5.0). A phylogenetic tree was constructed using the Neighbor-Joining (NJ) [31], Maximum Likelihood (ML) [32], and Minimum Evolution (ME) methods to ascertain the taxonomic position of strain L2-2. The complete genome sequence, 16S rRNA gene, and genomic maps of reference strains have all been submitted to the NCBI database.

### 2.3. Genome Sequencing and Analysis

The genome of strain L2-2 was sequenced using a de novo approach. Paired-end sequencing (PE150) of a ~400 bp insert library was performed on the Illumina platform. To enhance assembly quality, both second-generation (Illumina) and third-generation (PacBio) sequencing technologies were utilized. Quality control was performed on the sequenced data, and the assembly effectiveness was evaluated, including the number of contigs, scaffold N50 (bp) length, and completeness. Original paired-end reads were aligned to the assembled contigs using SOAP software (version 2.04) to assess base depth distribution. A sliding window analysis with various window sizes (1 kb, 3 kb, 5 kb, 8 kb, 10 kb) was conducted to calculate the average sequencing depth and GC content across the genome, thereby evaluating homogeneity and potential contamination. Contamination analysis was also performed using K-mer statistics. The Illumina data were assembled and optimized using SOAPdenovo2 (http://soap.genomics.org.cn/soapdenovo.html, accessed on 10 June 2024) with multiple K-mer values, and error correction and gap filling were conducted using Pilon (version 1.24). 

Coding sequences (CDSs) were predicted and annotated using Glimmer (http://ccb.jhu.edu/software/glimmer/index.shtml, accessed on 28 July 2024), GeneMarkS, and Prodigal. The GC content of the genome was calculated, and initial genome annotation was performed using NCBI’s Prokaryotic Genome Annotation Pipeline (PGAP). (https://www.ncbi.nlm.nih.gov/genome/annotation_prok, accessed on 20 August 2024).

To identify carbohydrate-active enzyme (CAZyme) genes, genome annotation was conducted utilizing the CAZy database (http://www.cazy.org/, accessed on 13 September 2024) [33]. Chitinase-related enzyme families were annotated, and potential enzyme subcellular localization was predicted. Signal peptides were identified using SignalP (http://www.cbs.dtu.dk/services/SignalP, accessed on 27 September 2024) [34], transporter proteins were determined through comparison with the TCDB database (http://www.tcdb.org/, accessed on 10 October 2024), and transmembrane domains were forecasted using TMHMM (http://www.cbs.dtu.dk/services/TMHMM/, accessed on 8 November 2024).

### 2.4. Genome-Based Phylogenetic Analysis

Thirty-one conserved housekeeping genes, including dnaG, frr, infC, nusA, pgk, pyrG, rplA, rplB, rplC, rplD, rplE, rplF, rplK, rplL, rplM, rplN, rplP, rplS, rplT, rpmA, rpoB, rpsB, rpsC, rpsE, rpsI, rpsJ, rpsK, rpsM, rpsS, smpB, and tsf, were selected as the foundation for phylogenetic analysis through genome alignment. Nineteen reference strains demonstrating the highest species-level similarity to strain L2-2 were retrieved from genome databases. Phylogenetic trees were constructed employing the Neighbor-Joining (NJ), Maximum Likelihood (ML), and Minimum Evolution (ME) methods using MEGA version 5.0. From these trees, seven species most closely related to strain L2-2 were identified for comparative genomic analysis.

Average Nucleotide Identity (ANI) and Average Amino Acid Identity (AAI) values were calculated using the ANI/AAI-Matrix tool available on the Kostas Lab Tools platform (http://enveomics.ce.gatech.edu/, accessed on 5 June 2024). To facilitate taxonomic classification, digital DNA-DNA hybridization (dDDH) values between strain L2-2 and the six reference genomes were determined via the Genome-to-Genome Distance Calculator (GGDC, https://ggdc.dsmz.de/ggdc.php, accessed on 5 June 2024).

### 2.5. Physiological, Biochemical, and Morphological Characterization

Strain L2-2 was inoculated onto a solid chitin medium (using chitin as the sole carbon source) and incubated at 30 °C for 7 days. Colony morphology was observed and recorded, including diameter, shape, texture, color, and margin characteristics.

For growth curve analysis, strain L2-2 was inoculated into 50 mL of LB broth (containing 10.0 g/L tryptone, 5.0 g/L yeast extract, and 10.0 g/L NaCl) with the initial OD_600_ adjusted to approximately 0.01. Cultures were incubated at 30 °C with shaking at 180 rpm for 72 h. Every 4 h, 2 mL of culture was sampled, and the OD_600_ value was measured using a UV-visible spectrophotometer (Model TN5000, Shanghai Youke Instrument Co., Ltd., Shanghai, China). Triplicate experiments were performed to ensure data reliability. Catalase activity was tested using a 3% (*v/v*) hydrogen peroxide solution. Gram staining was conducted to determine the Gram reaction type.

The API 50CH test kit and API 50CHB medium (bioMérieux, Beijing, China) were used to assess carbon source utilization and other biochemical properties. After culturing the strain in LB broth at 30 °C for 2 days, cell morphology was examined via scanning electron microscopy (SEM), while reproductive characteristics and spore formation were observed under a light microscope. Enzyme activity assays were performed for the following: amylase, oxidase, gelatinase, DNase, phosphatase, lipase, and urease. Additionally, biochemical reactions were evaluated using the following tests: indole production, Voges–Proskauer (V–P) test, nitrate reduction, hydrogen sulfide (H_2_S) production, lysine decarboxylation, and phenylalanine deamination.

### 2.6. Optimization of Fermentation Conditions

Fermentation conditions, including the type and concentration of carbon and nitrogen sources, significantly impact the enzyme-producing capacity of bacteria [35]. To enhance the chitinase yield of strain L2-2, the strain was inoculated into LB liquid medium at a 4% inoculation volume and cultured at 30 °C for 5 days. Enzyme activity was determined using the DNS method. A single colony of L2-2 on the LB solid plate was inoculated into 25 mL of LB liquid medium and cultured until the logarithmic growth phase to obtain the seed culture. In this study, a single-factor experimental design was employed [36] to systematically evaluate the effects of the following parameters on enzyme production: carbon source types (colloidal chitin, glucose, soluble starch, powdered chitin, sucrose, N-acetylglucosamine, α-lactose); carbon source concentrations (5, 10, 15, 20, 25, 30, 35 g/L); nitrogen source types (peptone, ammonium chloride, potassium nitrate, sodium nitrate, ammonium sulfate, yeast extract, beef extract, tryptone); nitrogen source concentrations (1, 2, 3, 4, 5, 6, 7 g/L); initial culture temperature (20, 25, 30, 35, 40 °C); initial pH values (4, 5, 6, 7, 8, 9); inoculum volume ratio (*v*/*v*, mL/mL: 2%, 4%, 6%, 8%, 10%, 12%); shaking speed (160, 180, 200, 220, 240 rpm); and fermentation duration (1, 2, 3, 4, 5, 6, 7 days).

Based on the optimization results of single-factor experiments, Plackett-Burman experimental design was performed using Design-Expert 13.0 software [37]. With chitinase activity (Y) as the response value, seven factors were selected: glucose concentration (A), ammonium sulfate concentration (B), culture time (C), culture pH (D), culture temperature (E), inoculum size (F), and shaking speed (G), with three parallel replicates per group. The software was used to analyze the experimental results, establish a regression model, and screen the culture factors that significantly influenced chitinase production by strain L2-2. According to the results of Plackett-Burman experiments, steepest ascent experiments were conducted. Based on the positive and negative effects of each factor on the strain’s enzyme production capacity, appropriate step sizes and climbing directions were designed, while other factors were set to the optimal conditions determined by single-factor optimization. Each group was repeated three times to determine the central point for response surface methodology (RSM) experiments. Based on the screened significant factors and the determined central point, a 3-factor 3-level Box–Behnken design was adopted, consisting of 17 groups in total. The low, medium, and high levels of each factor were denoted as “−1”, “0”, and “+1”, respectively. Using chitinase activity (U) of L2-2 as the response value, Design-Expert 13.0 software was employed to analyze the experimental data, establish a quadratic polynomial regression equation between enzyme activity and ammonium sulfate concentration (A), culture time (B), and culture pH (C), and perform reliability analysis and analysis of variance (ANOVA) on the model to determine the optimal A, B, and C conditions for enzyme production. Each group was repeated three times.

Fermentation culture was conducted according to the optimal conditions predicted by RSM to verify the accuracy of the model prediction. Chitinase activity was measured in three replicates to finally obtain the optimal enzyme-producing culture system for strain L2-2.

## 3. Results

### 3.1. General Characteristics of Strain L2-2

**Figure 1:**
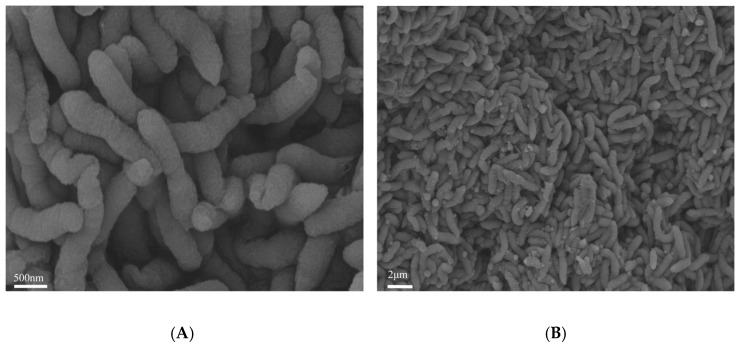
Morphological observation of strain L2-2 using scanning electron microscopy (SEM). Cells were cultured in LB medium at 30 °C for 2 days. Images were captured at magnifications of ×20,000 (**A**) and ×5000 (**B**).

**Figure 2:**
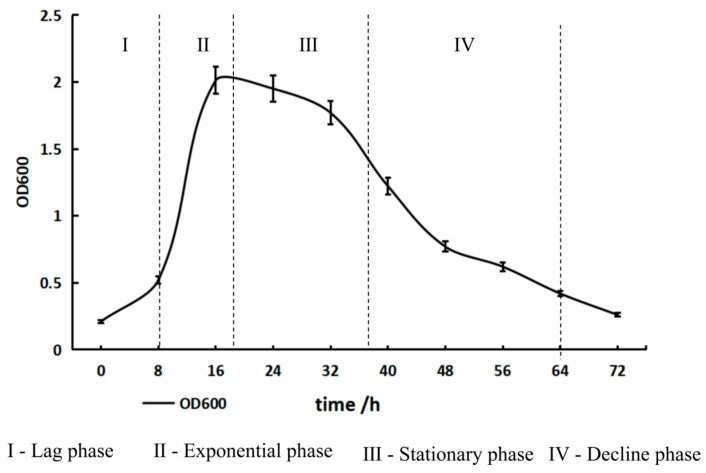
Growth curve of strain L2-2 in liquid LB medium for 72 h. The number of biological replicates in the growth curve determination experiment is 3.

Cells of the strain L2-2 are rod-shaped, measuring approximately 0.4–0.5 μm in diameter and 3.0–4.0 μm in length. They are non-spore-forming and motile. The strain is Gram-negative and reproduces through binary fission (Figure 1). After culturing on a chitin solid medium at 30 °C for 7 days under constant temperature, the strain formed colonies with a diameter of approximately 1 mm. The colonies were grayish-white, circular and flat, with a moist and glossy surface, soft texture, difficult to pick up, and no obvious odor. Observations under a light microscope confirmed the absence of motility and spore formation. The growth curve of strain L2-2 cultured in liquid LB medium for 72 h showed that the bacteria entered the logarithmic growth phase after 8 h, exhibiting a rapid growth rate. After 40 h, they transitioned into the decline phase (Figure 2).

**Figure 3:**
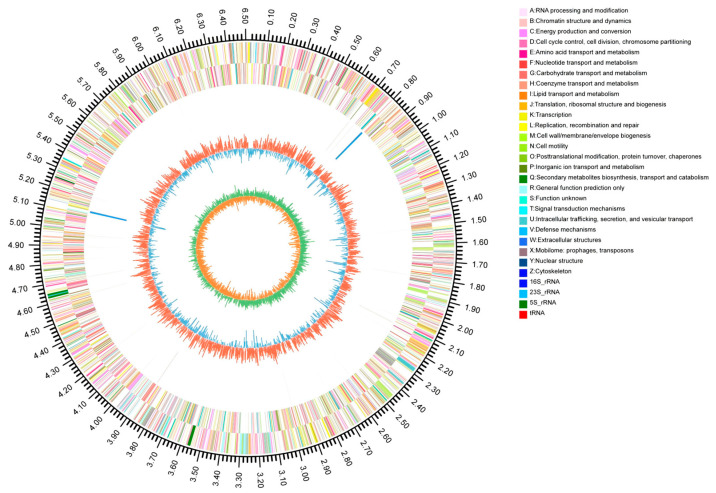
Circos Genome Circle Diagram of the L2-2 strain. The information corresponding to the outer to inner circles is genome size identification, gene information on the positive and negative strands, ncRNA, GC content, and GC Skew.

Whole-genome sequencing of strain L2-2 was performed using a combination of second-generation (Illumina) and third-generation (PacBio) sequencing technologies, resulting in a Circos genome circle map (Figure 3). The assembled genome has been submitted to the NCBI database under the accession number PRJNA1262638. The clean data for each sample exceeded 1.39 Gb, with over 94% of bases at Q30 quality, and a sequencing depth of 54.09×. The genome size is 6.512 Mb, featuring a G + C content of 68.6 mol%, and no plasmids were detected. Genome completeness is at 100%, with no loss of key marker genes; the contamination rate is low at 1.23% (<5%), indicating high assembly purity and the absence of significant heterogeneity. A total of 12 rRNA genes (including four each of 16S, 23S, and 5S rRNA), 63 tRNA genes (covering all 21 amino acids), and 21 sRNA genes were predicted. Additionally, 5643 coding sequences (CDS) were annotated.

### 3.2. Phylogenetic Analysis

A partial 16S rRNA gene sequence (1429 bp) of strain L2-2 was obtained through PCR amplification, and the full-length sequence (1520 bp) was retrieved from whole-genome annotation and submitted to NCBI (accession number: PV495750). BLAST analysis of the 16S rRNA gene sequence against the NCBI GenBank database revealed high similarity with *Roseateles depolymerans* JZY3-28^T^, *Roseateles noduli* HZ7^T^ (formerly *Mitsuaria noduli*), *Mitsuaria* sp. CR3-06, and *Mitsuaria* sp. K1, with sequence identities of 99.79%, 99.51%, 99.86% and 99.79%, respectively. The similarity between L2-2 and members of the genus *Roseateles* exceeds 98.7%, indicating that L2-2 likely belongs to this genus.

**Figure 4:**
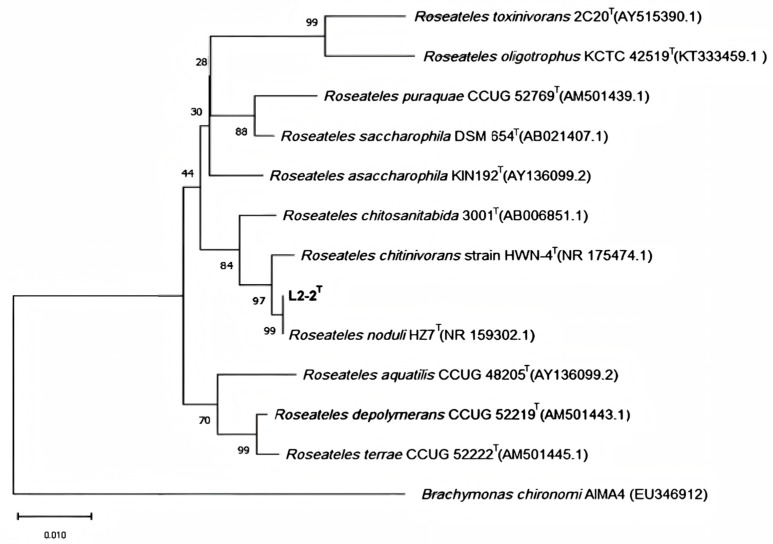
Phylogenetic tree of strain L2-2 based on 16S rRNA gene sequences. The tree was constructed using the neighbor-joining (NJ) method. Bootstrap values (expressed as percentages of 1000 replicates) are shown at the branch points. Evolutionary distances were computed using the method. The scale bar indicates the number of substitutions per nucleotide position.

A phylogenetic tree based on 16S rRNA gene sequences indicated that strain L2-2 clustered with other *Roseateles* species, with sequence identities generally exceeding 99% (Figure 4). A phylogenomic tree constructed using 31 conserved housekeeping genes further suggested that strain L2-2 is most closely related to *Roseateles noduli* HZ7^T^, supporting its classification within the genus *Roseateles*. To further confirm whether strain L2-2 is a member of the genus *Roseateles*, a comparative genomic analysis was conducted between strain L2-2 and the named species within this genus, which were conducted against six validly published type strains: *Roseateles noduli* HZ7^T^ (NIOE00000000), *Roseateles chitinivorans* HWN-4^T^ (PEOG00000000), *Roseateles chitosanitabida* 3001^T^ (BCYP00000000), *Roseateles aquatilis* CCUG 48205^T^ (NIOF00000000), *Roseateles depolymerans* CCUG 52219^T^ (CP013729.1), and *Roseateles terrae* CCUG 52222^T^ (NIOG00000000). The ANI values between L2-2 and these reference strains ranged from 79–95%, AAI values from 80–95%, and dDDH values from 23.2–63.2% (see Table 1). All values were below the widely accepted species delineation thresholds: ANI ≥ 95–96% [38], AAI ≥ 95–96% [39], and dDDH ≥ 70% [40]. Although strain L2-2 exhibits the highest similarity to *Roseateles noduli* HZ7^T^, the comparison result of TCS genes, which is below the cutoff (<0.98), aids in defining the genetic relationship between L2-2 and *Roseateles noduli* HZ7^T^. Additionally, there are significant phenotypic differences between strain L2-2 and its closely related strains in terms of morphological characteristics, size, growth traits, and colony color. Scanning electron microscopy reveals that L2-2 and *Roseateles noduli* HZ7^T^ differ significantly in morphology; specifically, the cell size of L2-2 (0.4–0.5 × 3.0–4.0 μm) is considerably larger than that of *Roseateles noduli* HZ7^T^ (0.3 × 1.5–2 μm). Coupled with their biochemical differences, these strains can be clearly distinguished from each other.

Thus, strain L2-2 can be considered a member of *Roseateles* distinct from *R. noduli* HZ7^T^.

### 3.3. Physiological and Biochemical Characterization

To comprehensively characterize strain L2-2, systematic physiological and biochemical analyses were conducted and compared with six closely related species within the genus *Roseateles* (see Table S1). The observation of bubble formation in 3% hydrogen peroxide indicated positive catalase activity in the strain.

Standard physiological and biochemical tests revealed that strain L2-2 tested positive for oxidase, nitrate reductase, lysine decarboxylase, phenylalanine deaminase, phosphatase, aesculinase, gelatinase, urease, H_2_S production, and indole production, confirming the presence of corresponding enzymatic activities. In contrast, the methyl red, Voges–Proskauer, DNase, and lipase tests yielded negative results.

Carbon source utilization tests showed that L2-2 could utilize malic acid, glucose, arbutin, trehalose, and raffinose as carbon sources (see Table S1). Results from the API 50CH kit demonstrated that strain L2-2 could ferment various sugars and their derivatives, including glycerol, erythritol, D-arabinose, L-arabinose, D-ribose, D-xylose, L-xylose, methyl-β-D-xylopyranoside, D-galactose, D-glucose, D-fructose, D-mannose, L-sorbose, L-rhamnose, inositol, D-mannitol, sorbitol, methyl-α-D-mannopyranoside, methyl-α-D-glucopyranoside, amygdalin, arbutin, aesculin ferric citrate, salicin, D-cellobiose, D-maltose, D-lactose, D-melibiose, D-sucrose, D-trehalose, inulin, D-melezitose, D-raffinose, starch, xylitol, D-turanose, D-lyxose, D-tagatose, D-fucose, L-fucose, D-arabitol, and L-arabitol.

Carbon sources that could not be utilized include citrate, N-acetylglucosamine, glycogen, D-gentiobiose, potassium gluconate, 2-ketogluconate, and 5-ketogluconate. A comprehensive analysis revealed that strain L2-2 shares certain similarities in carbon source utilization with its phylogenetic relatives but also exhibits distinct differences (see Table S1). For instance, L2-2 cannot utilize citrate, whereas *Roseateles noduli* HZ7^T^ can assimilate adipate and citrate but cannot utilize L-arabinose, galactose, sucrose, trehalose, or gentiobiose. In terms of enzymatic activity, strain L2-2 is capable of producing urease and hydrogen sulfide, and it can degrade tryptophan in peptone to generate indole. Additionally, its ability to ferment glycogen represents a unique metabolic trait of this strain.

In summary, these physiological and biochemical characteristics provide strong support for distinguishing strain L2-2 from *R. noduli* HZ7^T^.

### 3.4. Genetic Basis of Chitin Degradation

To further investigate the chitin-degrading capability of strain L2-2, whole-genome functional annotation and subcellular localization prediction were conducted, with a focus on the composition of carbohydrate-active enzyme (CAZyme) families [41]. These include six major protein families: glycoside hydrolases (GHs), glycosyltransferases (GTs), polysaccharide lyases (PLs), carbohydrate esterases (CEs), carbohydrate-binding modules (CBMs), and auxiliary activities (AAs). Within the GH family, the predicted gene products of GH1, GH2, and GH3 possess β-glucosidase activity and are predicted to contain signal peptides and transmembrane domains, suggesting a cytoplasmic localization. GH109 exhibits α-N-acetylgalactosaminidase activity, with no detectable signal peptide or transmembrane structure (Table 2). GH16, which has xyloglucan transferase activity, is predicted to contain both signal peptides and transmembrane domains; five coding sequences (CDSs) for this family were annotated.

Key families associated with chitin degradation—specifically GH18, GH23, and GH46—were also identified. As the primary family of bacterial chitinases, GH18 was represented by five annotated genes in the L2-2 genome, all encoding endo-β-N-acetylglucosaminidase with varying amino acid lengths. Gene0557 contains a Tat/SPI signal, while Gene3878 and Gene3879 possess Sec/SPI signal peptides, indicating potential secretion via specific pathways. In the GH23 family, five genes were annotated: Gene2651 and Gene2697 contain Tat/SPI signals, and Gene2882 and Gene3599 bear Sec/SPI signals. Two genes were identified in the GH46 family, with Gene3381 harboring a Sec/SPI signal. These localization predictions suggest that most of these enzymes are likely secreted proteins involved in extracellular chitin degradation. Gene3878 and Gene3879 are separated by 58 base pairs and share the same transcriptional direction, suggesting that they may belong to the same operon. Other genes are not clustered.

Strain L2-2 can grow on basal medium with chitin as the sole carbon source, confirming its ability to secrete chitinase extracellularly. A comparative analysis of chitinase sequences between L2-2 and its close relatives revealed that the amino acid sequences of chitinases from L2-2, *Mitsuaria* sp. H24L5A, and *Roseateles noduli* HZ7^T^ are all 680 amino acids in length (Table 2). In contrast, *Roseateles chitinivorans* HWN-4T, *Roseateles chitosanitabida* 3001^T^, and *Mitsuaria* sp. PDC51 have sequences of 679 amino acids, *Roseateles aquatilis* CCUG 48205^T^ has 661 amino acids, and *Mitsuaria* sp. 7 has a sequence length of 686 amino acids.

According to existing literature (e.g., phylogenetic analysis of *Mitsuaria chitinivorans* sp. nov.), a neighbor-joining tree constructed based on chitinase sequences shows that former *Mitsuaria* species form a robust clade (bootstrap > 96%), confirming the species-specificity of their chitinases (see Supplementary Figure S1). Their primary and secondary structures commonly feature the conserved GH18 motif DXDXE (located at amino acids 463–470), confirming their classification within the GH18 family.

Furthermore, the main products of colloidal chitin degradation by chitinase (Chi) secreted by L2-2 include N-acetyl-D-glucosamine, chitobiose, and chitotriose. This enzyme exhibits typical endo-chitinase activity, randomly cleaving chitin polysaccharide chains and ultimately generating oligosaccharides, chitobiose ((GlcNAc)_2_), and a small amount of monomeric GlcNAc. In summary, strain L2-2 possesses a complete set of chitinase-encoding capabilities at the genomic level. When combined with its enzyme secretion characteristics and actual degradation performance, this further supports its classification as a *Roseateles* genus bacterium with unique chitin-degrading functions.

### 3.5. Optimization of Fermentation Conditions for Enzyme Production

**Figure 5:**
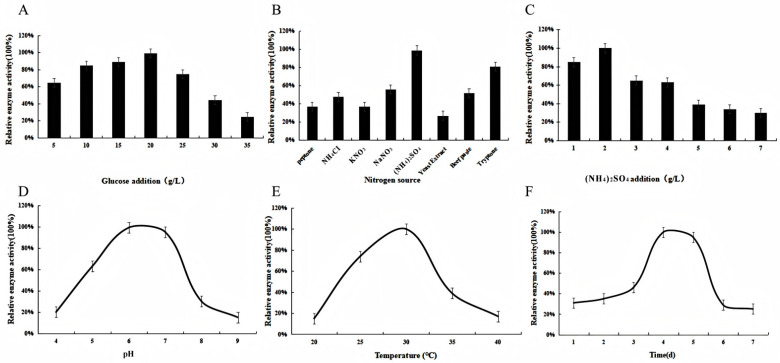
Effects of various fermentation conditions on the enzymatic activity of strain L2-2. (**A**) glucose concentration, (**B**) nitrogen source type, (**C**) ammonium sulfate concentration, (**D**) initial pH, (**E**) incubation temperature, (**F**) cultivation time.

In the screening of carbon sources, glucose significantly boosted the relative activity of chitinase, with the optimal concentration being 20 g/L (see Figure 5A, *p* < 0.0001). Among nitrogen sources, ammonium sulfate resulted in the highest enzyme activity (see Figure 5B, *p* < 0.0001), with the optimal concentration at 2 g/L (see Figure 5C, *p* < 0.0001). pH also had a significant effect on enzyme production: relative enzyme activity was greater at pH 6 or 7 than at pH 4, 5, 8, or 9 (see Figure 5D, *p* < 0.0001). Chitinase activity peaked at a cultivation temperature of 30 °C; beyond this temperature, the growth of strain L2-2 in the fermentation medium slowed considerably, and enzyme activity decreased sharply (see Figure 5E, *p* < 0.0001). Regarding the inoculum volume, 2% was determined to be the optimal rate, with enzyme activity decreasing as the inoculum size exceeded this level. Investigation into the fermentation duration revealed that maximum enzyme activity was achieved on day 4, followed by a slight decline after day 5, indicating 4 days as the optimal cultivation period (see Figure 5F, *p* < 0.0001). The definition of enzyme activity unit (U) is as follows: one enzyme activity unit is the amount of enzyme required to produce 1 μmol of reducing sugar per minute at 30 °C.

A Plackett-Burman design was used for preliminary screening of influencing factors (see Table S2). Regression analysis of enzyme production data was performed using Design-Expert 13.0 software, and the model exhibited a highly significant regression (*p* < 0.01), indicating strong statistical relevance. Analysis of *p*-values revealed that ammonium sulfate concentration (A), fermentation time (C), and pH (D) had extremely significant effects on chitinase production by L2-2 (*p* < 0.01), while glucose concentration also exerted a significant impact (*p* < 0.05). T-value analysis within the model demonstrated that glucose concentration, ammonium sulfate concentration, fermentation time, pH, and temperature had positive effects on enzyme production, whereas inoculum size had a negative effect (see Table S3).

**Figure 6:**
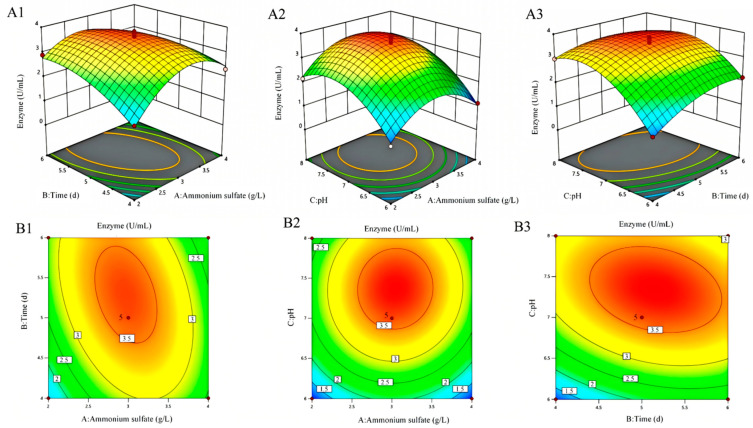
The comprehensive response surface plot illustrates the interaction effects of ammonium sulfate concentration, culture time, and culture pH on enzyme activity. Among them, (**A1**–**A3**) represent 3D plots of interaction effects, while (**B1**–**B3**) denote contour plots of interaction effects. The comprehensive response surface plot consists of three response surface plots, which sequentially correspond to the following variable combinations for the interaction effects on enzyme activity from left to right: (1) ammonium sulfate concentration (g/L) and culture time (d); (2) ammonium sulfate concentration (g/L) and culture pH; (3) culture time (d) and culture pH. (**A1**) 3D plot of the interaction effect of ammonium sulfate concentration (g/L) and culture time (d) on enzyme activity; (**A2**) 3D plot of the interaction effect of ammonium sulfate concentration (g/L) and culture pH on enzyme activity; (**A3**) 3D plot of the interaction effect of culture time (d) and culture pH on enzyme activity; (**B1**) contour plot of the interaction effect of ammonium sulfate concentration (g/L) and culture time (d) on enzyme activity; (**B2**) contour plot of the interaction effect of ammonium sulfate concentration (g/L) and culture pH on enzyme activity; (**B3**) contour plot of the interaction effect of culture time (d) and culture pH on enzyme activity.

Subsequently, three significant factors—ammonium sulfate concentration, fermentation time, and pH—were selected for a steepest ascent experiment. Results indicated that enzyme activity was highest under the conditions of 3 g/L ammonium sulfate, pH 7, and 5 days of cultivation. These values served as the central point for a Box–Behnken response surface design (see Figure 6). With enzyme activity (U) as the response variable, a total of 17 experiments were conducted involving the three factors at three levels each (see Table 3), and the following regression equation was established: U = 3.58 + 0.0634A + 0.2286B + 0.6514C − 0.4352AB + 0.0593AC − 0.2769BC − A^2^ − 0.4064B^2^ − 0.8744C^2^.

The model predicted the optimal fermentation conditions as follows: ammonium sulfate concentration of 3.092 g/L, fermentation time of 4.4 days, and pH of 7.1, with a predicted enzyme activity of 3.43 U/mL. In experimental validation, three parallel fermentation trials yielded enzyme activities of 3.34, 3.46, and 3.56 U/mL, respectively, all close to the predicted value, confirming the model’s reliability. Compared with the initial enzyme activity, the optimized conditions resulted in an approximately 24-fold increase (*p* < 0.0001) in enzyme activity.

## 4. Discussion

This study successfully isolated a strain L2-2 with chitin-degrading ability and reducing sugar production from the intestine of *Odorrana margaretae*. Comprehensive evaluations including phylogenetic analysis, whole-genome comparison, chemotaxonomy, and physiological-biochemical characteristics consistently showed that this strain belongs to the genus *Roseateles*.

The phylogenetic tree constructed based on 16S rRNA gene sequences indicated that strain L2-2 is closely related to known species of the genus *Roseateles*, with high phylogenetic support values (bootstrap values > 55%) and sequence similarities exceeding 99.0%, providing important support for the accuracy of its taxonomic status. Further analysis of the branching position of the phylogenetic tree constructed from chitinase sequences revealed that L2-2 is located between *Roseateles noduli*, *Roseateles chitinivorans*, and *Roseateles chitosanitabida*, suggesting that L2-2 is phylogenetically closer to the former *Mitsuaria* genus and has a closer genetic relationship with strains of this genus. The most prominent feature of this strain is its ability to grow using chitin as the sole carbon source, which is relatively rare among *Roseateles* strains, highlighting its unique ecological adaptability and metabolic potential.

At the phenotypic level, strain L2-2 showed significant differences from its close relatives in morphology, size, growth characteristics, and colony color. Scanning electron microscopy observations showed remarkable morphological differences between L2-2 and *Roseateles noduli*. The cell size of L2-2 is 0.4–0.5 × 3.0–4.0 μm, significantly larger than that of its close relative *Roseateles noduli* (0.3 × 1.5–2 μm). Physiologically and biochemically, L2-2 also exhibited obvious differences from six known close relatives of the genus *Roseateles* in carbon source utilization spectra, enzyme activity characteristics, and physiological metabolic capabilities. In particular, its chitin degradation ability and corresponding enzyme activity showed significant advantages, further enhancing its uniqueness as a new species and providing more sufficient basis for its taxonomic status.

Genomic annotation further revealed the genetic basis of chitin degradation in L2-2, identifying multiple chitinase-related enzyme families, including GH18, GH23, and GH46 families, which are key catalytic families for chitin decomposition [42]. Some enzymes were predicted to have signal peptides and transmembrane domains, indicating that they can be secreted extracellularly to directly act on chitin substrates [43]; this secretion mechanism is crucial for microbial competition and the degradation of complex organic matter in natural environments [44]. Amino acid sequence analysis showed that the Chi enzyme encoded by L2-2 is an endochitinase that can cleave glycosidic bonds within chitin chains. In addition, analysis of colloidal chitin degradation products confirmed that the chitinase of L2-2 mainly produces N-acetyl-D-glucosamine, chitobiose, and chitotriose (see Supplementary Figure S2), laying a foundation for subsequent enzyme function research and industrial applications. Antifungal experiments showed that the enzyme solution of L2-2 inhibits Rhizoctonia solani, suppresses fungal mycelial growth, and causes mycelial discoloration and senescence (see Supplementary Figure S3). This indicates its application potential in targeted biological control of plant diseases caused by this fungus, which is expected to reduce pesticide use and environmental pollution.

Furthermore, to explore the specific manifestation of the chitin degradation function of strain L2-2, the G18 family gene 3299 chitinase gene was studied [45]. By constructing the pET28a(+)-Chi vector and transforming it into *E. coli* BL21(DE3), after induction with 0.5 mmol/L IPTG at 30 °C for 16 h, the enzyme activity of the strain reached 5.4 U/mL; the concentration of the recombinant protein after treatment was 0.4 mg/mL. Enzyme kinetics analysis showed that its Km value was 6.2 mg/mL, and Vmax was 192 μmol/(min · mg). Preliminary analysis of the enzymatic properties of this recombinant enzyme showed that it has the best stability at around 35 °C, and high temperatures easily lead to changes in the enzyme structure and reduce activity; it has higher enzyme activity in citric acid-sodium citrate buffer at pH 4.0–6.0, which can be judged as an acidic chitinase, and it has better stability at around pH 6.0; meanwhile, metal ions such as Mn^2+^, Zn^2+^, and Na^+^ can promote enzyme activity, and appropriate concentrations can also enhance enzyme stability. This result not only verifies the function of the relevant chitinase gene in strain L2-2, but also provides clues for understanding the adaptation mechanism of this strain in the ecological environment. The exploration of enzyme activity conditions (such as temperature and pH) has laid a key foundation for maintaining the activity stability of Chi enzymes in different ecological scenarios, which is the core premise for exerting their ecological functions. For example, in acidic soils, using its optimal pH of approximately 6.0 to regulate the microenvironment can efficiently degrade chitin-containing organic matter, regulate the microbial community, and enhance soil ecological stability. It is particularly noteworthy that *Rhizoctonia solani*, as an important pathogen causing diseases in various crops, is prone to breeding and spreading in acidic soil environments. Based on the high activity of Chi enzyme under acidic conditions, if its inhibitory effect on this pathogen can be fully exerted in the future, it can greatly reduce the occurrence of crop diseases and agricultural losses. At the same time, the enzyme’s adaptability to temperature and response to metal ions provide important references for subsequent fermentation optimization. Combining these characteristics to further adjust the fermentation conditions is expected to improve enzyme activity and enhance its application potential in chitin waste treatment, agricultural biological control, and other aspects. In the future, we will clarify the relationship between chitinase activity and identified genes and increase production to further enhance its ecological function applications, laying a solid foundation for the subsequent development of its enzymatic properties and industrial application potential [46,47].

Finally, in the optimization of fermentation conditions, this study systematically screened the key factors affecting enzyme production through single-factor tests and response surface methodology. The results showed that pH was the most critical factor affecting chitinase yield, followed by fermentation time and ammonium sulfate concentration. The optimal pH not only promotes strain growth but also enhances enzyme expression and activity; precise control of fermentation time can ensure continuous metabolic activity; and ammonium sulfate, as a nitrogen source, can regulate protein synthesis and metabolic flux [48]. Under optimized conditions, the enzyme activity was significantly improved, reaching 3.46 U/mL, approximately 24 times that under unoptimized conditions. This improvement highlights the industrial potential of this strain and provides a solid experimental basis for the efficient biological conversion of environmental chitin resources [49].

## 5. Conclusions

In this study, a chitin-degrading bacterium, *Roseateles* sp. L2-2, was isolated from the intestine of *Odorrana margaretae* and validated as a novel species through phylogenetic and genomic analyses. Strain L2-2 displays distinct physiological and biochemical traits, demonstrating superior chitin degradation capacity and enzymatic activity compared to six closely related *Roseateles* species. Notable differences are observed in carbon source utilization, metabolic profiles, and cell morphology (e.g., rod-shaped cells measuring 0.4–0.5 × 3.0–4.0 μm, larger than congeneric strains). Genomic annotation identifies genes encoding chitinases (GH18, GH23, and GH46 families) and antibiotic biosynthesis pathways, endowing the strain with efficient chitinolytic activity and antifungal potential against Rhizoctonia solani. Under optimized fermentation conditions at 30 °C, its chitinase activity reaches 3.46 U/mL, representing a 24-fold increase from the initial level, which highlights its industrial potential as a high-yield producer of animal-derived chitinases. Ecologically, the strain facilitates host occupation of higher ecological niches by degrading insect exoskeleton chitin and maintains intestinal microbial stability via unique adaptive genes. It shows promising applications in chitin waste biodegradation, agricultural biocontrol, wastewater treatment, and environmental remediation. The purified strain has been deposited in the China Center for Type Culture Collection (CCTCC M 2024275), and its 16S rRNA and whole-genome sequences have been submitted to NCBI (accession numbers PV495750 and PRJNA1262638), providing a foundation for further research on enzymatic regulation mechanisms and industrialization of its biocatalytic functions.

## Figures and Tables

**Table 1:** Genome relatedness based on ANI, AAI, and dDDH values between L2-2 and closely related species of the genus *Roseateles*.

Close Related Species.	ANI (%)	AAI (%)	dDDH (%)
*Roseateles noduli* HZ7^T^	95	95	63.2
*Roseateles chitinivorans* HWN-4^T^	86	87	31.2
*Roseateles chitosanitabida* 3001^T^	84	88	28.3
*Roseateles aquatilis* CCUG 48205^T^	84	87	28.2
*Roseateles depolymerans* CCUG 52219^T^	79	80	23.3
*Roseateles terrae* CCUG 52222^T^	79	80	23.2

In conclusion, strain L2-2 is proposed as a new species within the genus *Roseateles*.

**Table 2:** Comparison of three chitinase families of the L2-2 from genome-wide annotation.

Family	Activity Rates in Family	Enzyme	Gene ID	Length (aa)	Protein Type Sec/SPI	
GH18	Chitinase endo-beta-N-acetylglucosaminidase	(EC 3.2.1.14) (EC 3.2.1.96)	0557	452	Tat	SPI
			1585	470	NA	NA
			3299	531	NA	NA
			3878	870	Sec	SPI
			3879	680	Sec	SPI
GH23	Chitinase lysozyme type G	(EC 3.2.1.14) (EC 3.2.1.17)	2651	204	Tat	SPI
			2697	283	Tat	SPI
			2882	274	Sec	SPI
			3599	242	Sec	SPI
			3978	297	NA	NA
			5071	238	NA	NA
GH46	Chitinase	(EC 3.2.1.14)	3381	492	Sec	SPI
			3489	223	NA	NA

NA: other protein type, meaning no signal peptide was annotated.

**Table 3:** Box–Behnken test results. (A), addition of (NH4)_2_SO_4_ (g/L); (B), Time (d); (C), pH value.

No.	A	B	C	Enzyme Activity (U·mL^−1^)
1	2	4	7	1.52
2	3	5	7	3.73
3	3	5	7	3.54
4	4	6	7	1.95
5	3	5	7	3.29
6	2	5	6	0.92
7	3	4	8	3.00
8	3	5	7	3.78
9	2	5	8	2.16
10	3	6	6	2.16
11	3	5	7	2.63
12	4	4	7	2.32
13	4	5	8	2.61
14	3	6	8	2.85
15	4	5	6	1.13
16	3	4	6	1.19
17	2	6	7	2.90

*p* < 0.01, highly significant; lack-of-fit term *p* > 0.05, not significant; R^2^ = 0.9819, R^2^Adj = 0.9587, Adeq Precision = 17.2803.

## Data Availability

The data presented in this study are openly available from the NCBI in the BioProject PRJNA1262638.

## Supplementary Materials

The following supporting information can be downloaded at: https://www.mdpi.com/article/10.3390/microorganisms13092033/s1, Figure S1: Phylogenetic tree of strain L2-2 based on chitinase enzyme sequences; Figure S2: Products of enzymatic hydrolysis of colloidal chitin; Figure S3: Antibacterial experiment of strain L2-2; Table S1: Comparisons of phenotypic characteristics between L2-2 and related species of the genus *Roseateles*; Table S2: Results of the Plackett-Burman test and ANOVA results; Table S3: Results of Steepest Climbing Test.
