# Supplementary material for: Characterization and Optimization of Fermentation Conditions of Roseateles sp. L2-2, a Novel Chitin-Degrading Bacterium from the Intestine of Odorrana margaretae
Source: Microorganisms. 2025 Aug 30;13(9):2033. doi: 10.3390/microorganisms13092033 (PMC12473003; doi:10.3390/microorganisms13092033)
Supplement: Supplementary file 1 [file microorganisms-13-02033-s001.zip › microorganisms-3767242-supplementary.pdf]

microorganisms

MDPI

Supplementary Materials

Table S1. Comparisons of phenotypic characteristics between L2-2 and related species of the genus *Roseateles*.

Characteristic	1	2	3	4	5	6	7
Isolation source	Intestinal	nodule	Water	Soil	Water	Water	Soil
Motility	+	+	+	+	+	+	+
Temperature(◦C)	20-40	4-45	10-37	20-34	20-30	5-43	10-37
pH	5-9	5-9	6.5-8.5	5-9	7	5-8	7
Morphology	rod-shaped	rod-shaped	rod-shaped	rod-shaped	rod-shaped	rod-shaped	rod-shaped
Colony colour	Gray	white	white	brown	white	pink	white
Oxidase	+	+	+	+	+	+	+
Urease	+	-	-	-	-	+	-
Nitrate reduction	+	-	+	+	-	-	-
Catalaseactivity	+	+	+	+	-	+	+
Gelatinhydrolysis	+	+	+	+	+	+	+
Hydrolysis of starch	+	-	+	W	-	N	+
N-Acetylglucosamine	+	+	+	-	-	-	-
D-Glucose	+	-	-	+	+	+	+
D-Fructose	+	-	-	+	-	+	+
D-Mannose	+	+	+	-	-	+	+
D-Mannitol	+	+	+	-	-	+	+
Gluconate	-	-	-	+	+	+	+
Trehalose	+	-	+	W	-	N	-
D-Serine	+	+	-	-	+	+	-
L-Phenylalanine	+	-	+	+	+	N	+
Inosine	+	-	+	+	+	+	-
Galactose	+	-	+	-	-	+	+

 https://doi.org/10.3390/microorganisms13092033

β-Galactosidase	+	-	-	W	-	+	+
Sorbitol	+	N	+	-	N	-	N
L-Malate acid,	+	N	+	+	-	-	W
glycogen	-	+	+	N	-	+	+
maltose	+	+	+	+	+	+	+
Citrate	-	N	+	N	-	-	W
G+C	68.6	68.5	69.2	69.3	68.8	66.2	66.4

Data from the present study and data for *Roseateles noduli* HZ7^{T} is from Fan et al ; Data for *Roseateles chitinivorans* HWN-4^{T} is from Sisinthy et al; Data for *Roseateles chitosanitabida* 3001^{T} is from Amakata et al ; Data for Roseateles aquatilis CCUG 48205^{T} and Roseateles terrae CCUG 52222^{T}are from Gomila et al; Data for*Roseateles depolymerans* CCUG 52219^{T} is fromSuyam et al . Strains: 1, L2-2; Strains: 2, *Roseateles noduli* HZ7^{T}; Strains: 3, *Roseateles chitinivorans* HWN-4^{T}; Strains: 4, *Roseateles chitosanitabida*3001^{T}; Strains: 5, *Roseateles aquatilis* CCUG 48205^{T}; Strains: 6, *Roseateles depolymerans* CCUG 52219^{T}; Strains: 7, *Roseateles terrae* CCUG 52222^{T}; +, Positive; -, negative; W, weak; N, data not available.

Table S2.1 Results of the Plackett-Burman test

**No.**	**level**							**Chitinase activity (U/mL)**
	**A**	**B**	**C**	**D**	**E**	**F**	**G**	
1	1	-1	-1	-1	1	-1	1	1.04
2	-1	1	1	-1	1	1	1	1.72
3	-1	-1	-1	1	-1	1	1	1.18
4	1	1	-1	-1	-1	1	-1	1.70
5	-1	-1	-1	-1	-1	-1	-1	1.03
6	-1	1	-1	1	1	-1	1	1.73
7	1	1	1	-1	-1	-1	1	1.77
8	1	-1	1	1	-1	1	1	2.08
9	-1	-1	1	-1	1	1	-1	1.42
10	1	-1	1	1	1	-1	-1	2.73
11	1	1	-1	1	1	1	-1	2.57
12	-1	1	1	1	-1	-1	-1	2.89

Table S2.2 ANOVA results

Factor	SS	df	MS	F-statistic	p-value	significance ranking
model	4.35	7	0.6211	26.87	0.0033**	
Glucose source (A)	0.3072	1	0.3072	13.29	0.0219*	5
Ammonium sulphate source (B)	0.7008	1	0.7008	30.32	0.0053**	3
Time of culture (C)	0.9409	1	0.9409	40.70	0.0031**	2
pH of culture (D)	1.69	1	1.69	73.00	0.001**	1
Temperature of culture (E)	0.0261	1	0.0261	1.13	0.3476	6
Amount of inoculation (F)	0.0225	1	0.0225	0.9748	0.3794	7
Speed of cultivation (G)	0.6627	1	0.6627	28.67	0.0059*	4

Note: $R^2 = 0.9792$, $R^2Adj = 0.9427$; "*" indicates significance ($P < 0.05$); "**" indicates extreme significance ($P < 0.01$)

Table S3 Results of Steepest Climbing Test

NO.	Ammonium sulphate (g/L)	Time (d)	pH	Chitinase activity (U/mL)
1	1	3	5	2.24
2	2	4	6	3.34
3	3	5	7	3.54
4	4	6	8	1.18
5	5	7	9	0.66

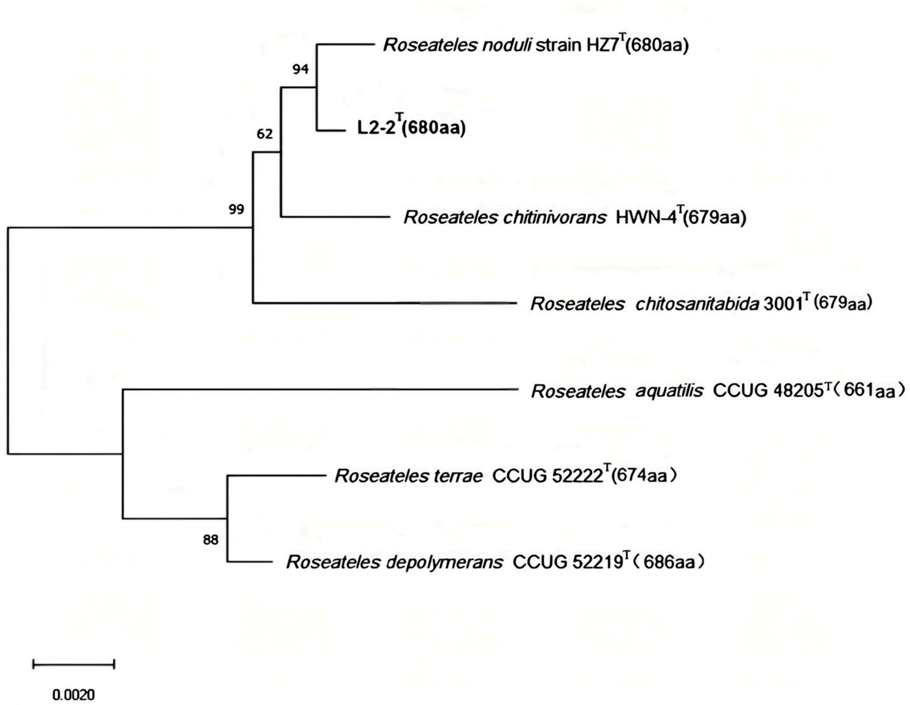

Figure S1. Phylogenetic tree of strain L2-2 based on chitinase enzyme sequences. The tree was constructed using the neighbor-joining (NJ) method.

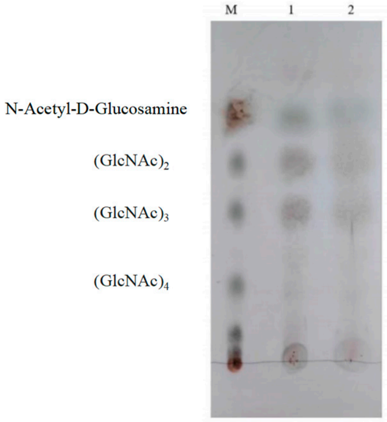

Figure S2. Products of enzymatic hydrolysis of colloidal chitin. Lane M: chito-oligosaccharide standards; Lanes 1 and 2: enzymatic hydrolysates after 12 hours of reaction.

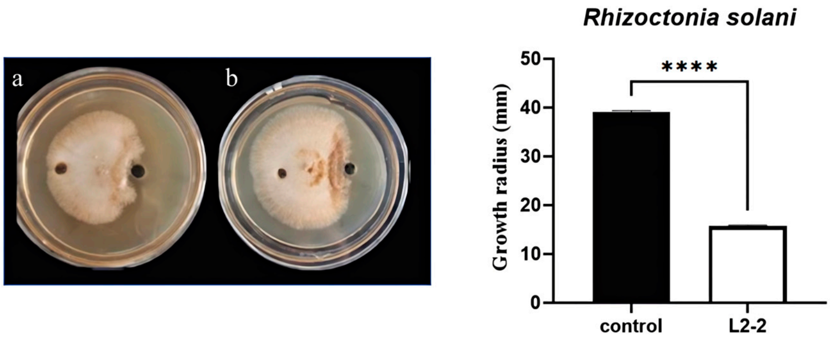

Figure S3. Antibacterial experiment of strain L2-2. The left figure shows "Analysis of the Antifungal Activity of the Chitinase Fermentation Broth from Strain L2-2 Against Rhizoctonia solani in 7d," and the right figure shows the statistics of the growth radius of Rhizoctonia solani hyphae under the influence of the control (sterile water) and L2-2 fermentation broth, with a P value < 0.0001.
